# The Versatility of the Roskamp Homologation in Synthesis

**DOI:** 10.3390/molecules30061192

**Published:** 2025-03-07

**Authors:** Margherita Miele, Aljoša Smajić, Vittorio Pace

**Affiliations:** 1 Department of Chemistry, University of Turin, Via P. Giuria 7, 10125 Turin, Italy; margherita.miele@unito.it; 2 Division of Pharmaceutical Chemistry, Department of Pharmaceutical Sciences, University of Vienna, Josef-Holaubek-Platz 2, A-1090 Vienna, Austria; aljosa.smajic@univie.ac.at

**Keywords:** homologation, Roskamp reaction, asymmetric reaction, α-diazocarbonyl compounds, Weinreb amides, α-silyl ketones

## Abstract

Modern organic synthesis continues to benefit from the flexibility of α-diazo carbonyl intermediates. In the context of homologation processes, the Roskamp reaction—first introduced in 1989—has become a valuable tool due to its selectivity and mild condition reactions for accessing important synthons amenable to further functionalization as β-keto esters. The fine-tuning of reaction parameters—including the nature of Lewis acids, solvents, and temperature—has enabled the development of catalyzed continuous-flow methodologies, as well as a series of asymmetric variants characterized by high transformation rates, excellent stereocontrol, and formidable chemoselectivity. This review aims to emphasize the attractive features of the Roskamp reaction and its applicability for addressing challenging homologation processes.

## 1. Introduction

**Figure 1:**
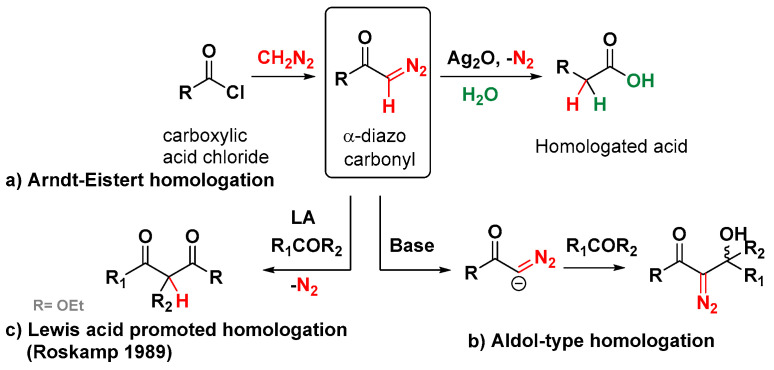
Main pathways of α-diazocarbonyl compound reactions with aldehydes and ketones [26].

**Figure 2:**
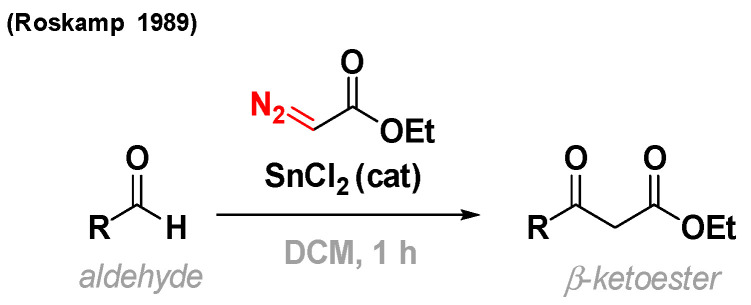
The Roskamp reaction [26].

Currently, homologation reactions represent an essential tool in organic and medicinal chemistry, considering the ever-increasing need for more complex compounds and pharmaceuticals. A homologation reaction is defined as the elongation of the carbon skeleton chain with a methylene group that is inserted into the molecule to obtain the higher homologue. This reaction can be applied to organometallic- [1,2,3,4,5,6,7,8,9,10,11] and radical-levered logics. In this context aldehydes and ketones, among others, such as Weinreb amides [12,13,14,15] and iso(thio)cyanates [16,17,18,19], are common substrates primed for undergoing homologation processes. One of the most popular C1-insertion protocols is the addition of diazo compounds, illustrated by the venerable Arndt–Eistert reaction (Figure 1a), in which diazomethane is used as a homologating agent, leading to an α-diazo ketone. Upon treatment with water in the presence of a catalyst, such as silver, platinum, or copper, the reaction eliminates N_2_, making the reaction clean and efficient [20]. The employment of α-diazocarbonyl compounds has a long history of useful applications in organic chemistry. They are easily prepared from readily accessible precursors and can be induced to undergo a wide variety of chemical transformations under very mild conditions. We can identify two broad categories of reactions of α-diazo-carbonyls with aldehydes and ketones: (1) an aldol-type addition promoted by a base, involving the retention of the diazo function (Figure 1b), and (2) a related process involving the loss of nitrogen and the formation of a β-dicarbonyl, which requires a Lewis acid (Figure 1c) [21].

In aldol-type additions, the diazocarbonyl precursor must be susceptible to ionization, promoted by a base, leading to an α-diazocarbonyl anion capable of reacting with aldehydes or ketones. For the deprotonation step, a dilute solution of potassium hydroxide in methanol or ethanol is the preferred combination [22]. However, LDA has been by far the most widely employed base for the deprotonation of diazocarbonyls, enabling the conversion of aldehydes and ketones into α-diazo-β-hydroxycarbonyl adducts, which are suitable for subsequent transformations into nitrogen-free products. An attractive example involves the catalytic action of rhodium(II) acetate, combined with the use of ethyl lithium acetate to afford β-keto ester compounds [23].

On the other hand, acid-promoted reactions of aldehydes and ketones lead directly to nitrogen-free β-dicarbonyl products, such as β-keto esters or β-diketones, producing nitrogen gas as a byproduct [24,25].

β-Keto esters represent pivotal multicoupling reagents in synthesis [27], characterized by the concomitant presence of both electrophilic and nucleophilic sites [28]. Accordingly, they serve as substrates for carbon–carbon bond formation operations with wide applicability across the chemical sciences, including agrochemicals, polymers, natural product synthesis, and pharmaceuticals [29]. For example, canonical drugs featuring pyridazine, barbiturate, and pyrazolone cores, among others, can be prepared through their judicious manipulation [30,31]. Furthermore, the use of β-keto esters as competent starting materials has more recently been applied to access Sumatriptan, Edaravone, and natural products such as Nonactin and Kermesic acid [32,33,34,35]. The Roskamp reaction, reported in 1989 [26], consisting of the Lewis acid-catalyzed reaction of alkyl diazoesters with aldehydes, is one of the most powerful and useful methodologies for synthesizing β-keto carbonyls and, in particular, β-keto esters. Today, it is a well-established yet underrated synthetic methodology, where direct conversion, high selectivity, mild conditions, and short reaction times make it a noteworthy subject for a comprehensive review of its chemistry and further applications in modern chemistry.

## 2. The Roskamp Reaction: A Powerful Method for the Synthesis of β-Keto Esters

The Roskamp reaction was first conceived for the conversion of aldehydes to alkenes via a pseudo-Wittig type reaction and quickly resulted in a selective and highly efficient method for the synthesis of β-keto esters.

With the aim of generating an alkylidene-type reagent by reacting ethyl diazoacetate with a low-valent main group metal, R. Holmquist and E. J. Roskamp observed that the reaction of aldehydes with diazocarbonyls in the presence of tin-(II) chloride produced a single product: a β-keto ester (Figure 2).

This method involves the nucleophilic addition of alkyl diazoacetates in the presence of 10 mol % of various Lewis acids, such as BF_3_, ZnCl_2_, AlCl_3_, SnCl_2_, GeCl_2_, and SnCl_4_. This reaction occurs readily in dichloromethane at or below room temperature (Figure 2), showing sensitivity toward aliphatic aldehydes (which are more reactive due to enolization), while aromatic aldehydes react much more slowly and require an additional catalyst [26].

**Figure 3:**
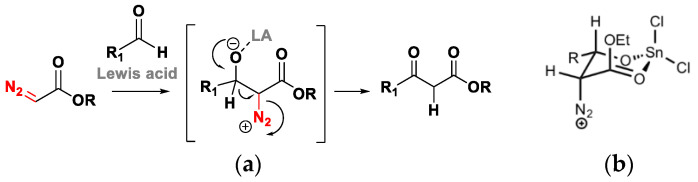
(**a**) Mechanism of the Roskamp reaction. (**b**) Transition state of the diazoacetate complex.

The mechanism of the reaction is likely to proceed via a tetrahedral intermediate that can undergo a rapid 1,2-hydrogen rearrangement (Figure 3b). Reagents like diazoacetate bear a negatively located charge adjacent to the diazo motif, allowing for nucleophilic attack under the employed conditions. The delocalization of electrons at the electron-withdrawing carbonyl group ensures further stabilization and makes this a safe method for obtaining the one-carbon homologation of carbonyl derivatives (Figure 3a).

Temperatures around −15 °C already favor the direct conversion of aldehydes into β-keto esters. Optimal yields were obtained employing Lewis acids like BF_3_, GeCl_2_, and SnCl_2_; various solvents, such as THF, DME, Et_2_O, CH_2_Cl_2_, and PhCH_3_, can also be used. Although CH_2_Cl_2_ yields the best results, the reactions conducted in ether, THF, DME, and CH_3_CN were slower and/or characterized by the formation of additional side products. However, in comparison to other related one-carbon homologation methods like Tiffeneau–Demjanov rearrangement, Buchner–Curtius-Schlotterbeck reaction, and Arnd–Eistert reaction, the Roskamp reaction appears stable under atmospheric conditions, allows for selectivity, and benefits from a mild environment [36,37,38,39,40,41,42,43].

**Figure 4:**
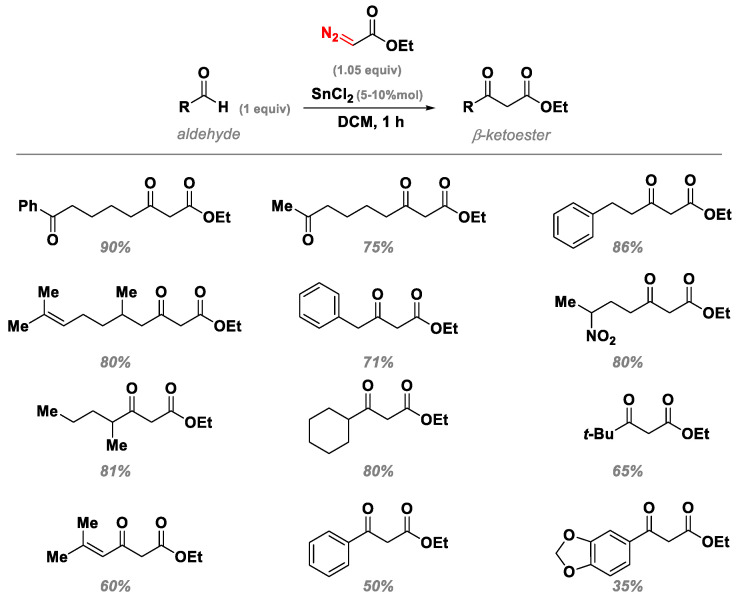
β-keto ester derivatives established by the Roskamp reaction.

The high conversion rates, as well as the broad substrate scope (Figure 4), make the Roskamp reaction one of the most popular and efficient methods for the synthesis of β-keto esters today.

Distinct metal catalysts were explored, including NbCl_5_ [44], Sc(OTf)_3_ [45], MoO_2_Cl_2_ [46], Al_2_O_3_ [47], and ZrCl_4_ [48], as well as Brønsted acids, such as HBF_4_-Et_2_O [49], and the use of sustainable and reusable heterogeneous catalysts, such as zeolites [50], montmorillonites [51], yttria-stabilized zirconia [52], and aluminosilicates [53,54,55].

**Figure 5:**
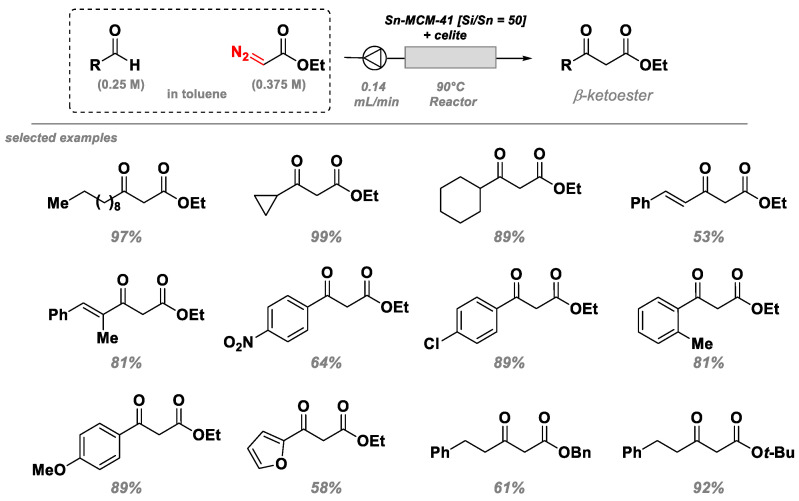
β-keto ester derivatives established using the process developed by Ishitani, Kobayashi, and coworkers.

With the increasing attractiveness of continuous-flow technologies adopted in both academia and industry, Ishitani, Kobayashi, and coworkers prepared β-ketoesters utilizing Sn-MCM-41 as a heterogeneous catalyst. The process is characterized by excellent yields, simple and safe operation, and broad functional group tolerance (Figure 5). Notably, some of these manifolds have been subsequently converted into bioactive pyrazoles and coumarins [56].

**Scheme 1:**
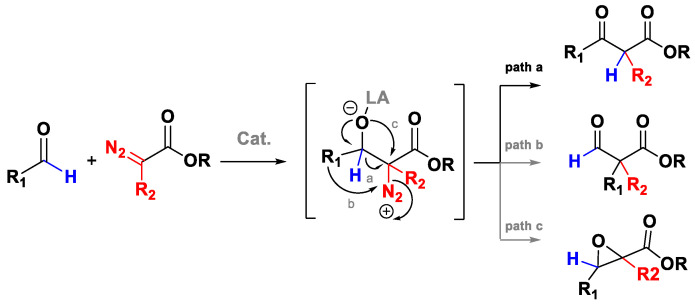
Different rearrangement pathways during Roskamp reaction: R = OEt, R_1_ = alkyl or aryl, R_2_ = H or alkyl.

While the Roskamp reaction has been thoroughly employed in synthesis [57,58,59], the development of asymmetric variants presented significant challenges as a consequence of the inherent difficulties in controlling the three possible (different) rearrangement pathways of the tetrahedral intermediate. Indeed, in the case of the undesired shift of the R_1_ group to the α-position, a β-formyl ester would be obtained (Scheme 1, pathway b). Similarly, the possibility of internal nucleophilic displacement, promoted by the alkoxide*,* yielding an epoxide would not be remote (Scheme 1, pathway c). Additionally, the kinetic instability of the stereogenic center, which is intrinsically primed for undergoing epimerization events should be taken into account [45].

## 3. A Strategy Toward Asymmetric Variants

### 3.1. Roskamp Reaction and Chiral Auxiliaries

**Scheme 2:**
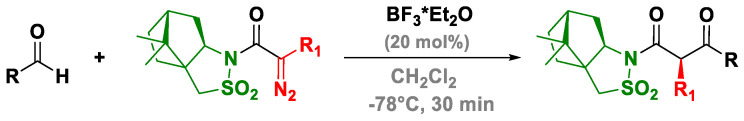
The Roskamp reaction in combination with chiral auxiliary at the α-diazocarbonyl moiety.

Maruoka, in 2009, reported an innovative procedure that allows for the desirable asymmetric C-C bond formation through the installation of a chiral auxiliary in the α-diazocarbonyl substrate [60]. As in the original Roskamp synthetic pathway, the aldehyde can undergo nucleophilic addition of the α-diazocarbonyl substrates in the presence of Lewis acids (Scheme 2). Indeed, different auxiliaries were screened, thus allowing for the deduction of critical structural characteristics of α-diazocarbonyl that control stereoselectivity.

**Scheme 3:**
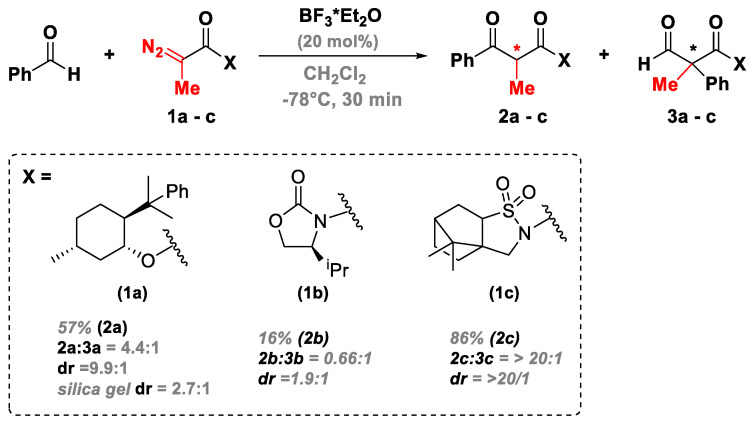
Chiral auxiliaries screened for the asymmetric Roskamp reaction.

First attempts using (−)-phenylmenthyl α-methyl-α-diazoacetate (**1a**) afforded the corresponding keto ester, which unfortunately gradually epimerized at the α-chiral center during isolation. Thus, chiral auxiliaries that impart a large steric hindrance to the products, which may offer a high kinetic barrier against enolization, like (*S*)-4-isopropyl-2-oxazolidinone (**1b**) and (−)-camphorsultam (**1c**), were tested (Scheme 3). While the reaction with **1b** furnished the corresponding α-methyl-β-keto imide in low yield, along with the concomitant formation of the epoxide, the use of **1c** led exclusively to the desired product with high diastereoselectivity and no erosion of the stereochemical information during isolation.

**Figure 6:**
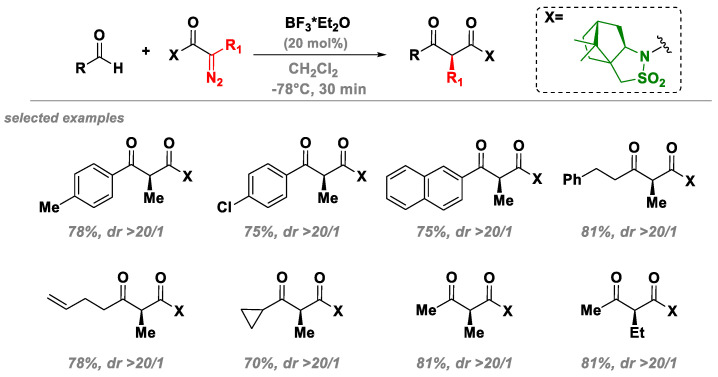
Selected examples of the Maruoka–Roskamp protocol.

According to the protocol of Maruoka, (−)-camphorsultam-containing diazocarbonyl compounds, in combination with a diverse set of functionalized aldehydes, provided the desired adducts with uniformly high selectivity and stereocontrol (Figure 6). It is important to note that the overall efficiency of the method also relies on the straightforward removal of the auxiliary group.

### 3.2. New Directions in Asymmetric Roskamp Reactions

**Scheme 4:**
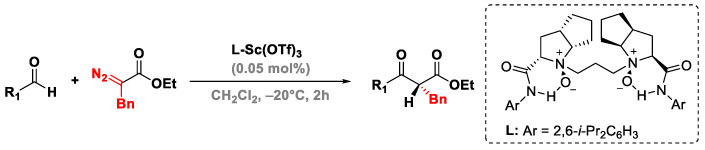
Roskamp–Feng reaction with L–ramipril acid–derived *N*,*N*′–dioxide. Ar = 2,6–i-Pr_2_C_6_H_3_.

In the pursuit of a more economically efficient and catalytic approach that does not require an additional removal step, Feng and coworkers introduced, in 2010, a catalytic asymmetric Roskamp reaction using chiral *N*,*N*′-dioxide-scandium(III) Lewis acid complexes (Scheme 4). The so-called Roskamp–Feng reaction involves ethyl α-benzyl α-diazoacetates reacting with benzaldehydes, facilitated by an *L*-ramipril acid-derived *N*,*N′*-dioxide in complex with Sc(OTf)_3_ under mild conditions.

**Figure 7:**
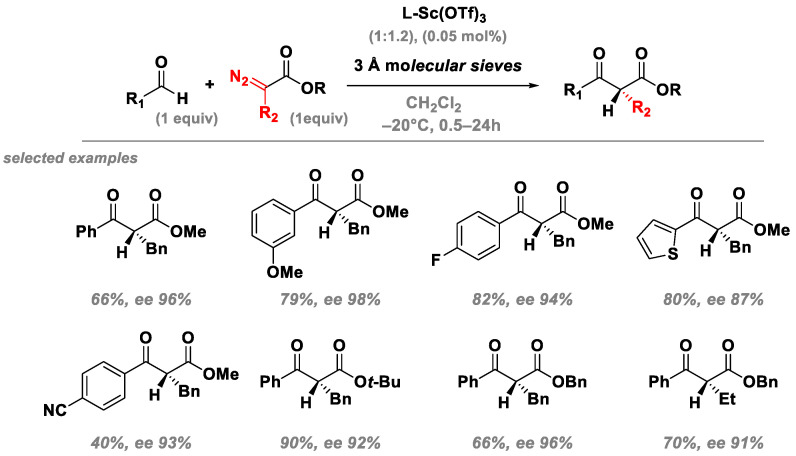
Feng’s modification of the Roskamp reaction.

As a result of the steric hindrance of the above-mentioned *N*,*N′*-dioxide-scandium(III) complex, the potential migration of the R_1_ group and the epoxide formation are suppressed, whereas the enantioselectivity and reactivity are remarkably increased (Figure 7). Moreover, the reaction was optimized using 3 Å molecular sieves with a catalyst loading of 0.05 mol %. Aromatic aldehydes bearing substituents with different electronic behaviors, as well as variable steric hindrance, were uniform (up to 98% enantioselectivity and up to 99% yield). However, aliphatic and α, β-unsaturated aldehydes were not optimal substrates for the protocol [45].

### 3.3. A Novel Lewis Acid Catalyst

**Figure 8:**
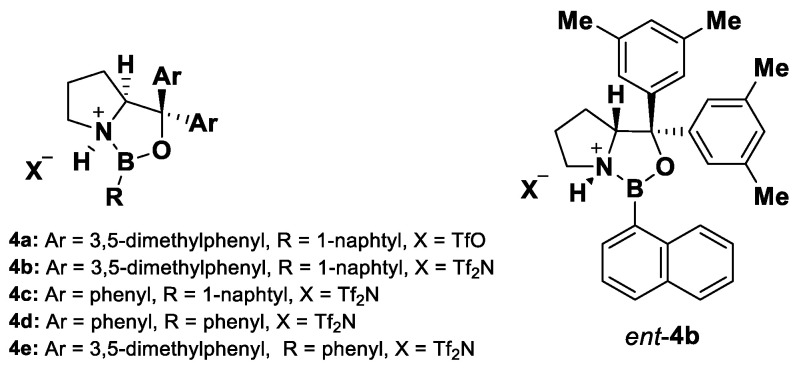
Oxazaborolidinium ion catalysts. Tf = trifluoromethanesulfonyl.

To overcome the issues that have emerged with aliphatic aldehydes [45], a new catalytic asymmetric Roskamp reaction was developed by Ryu and coworkers [61]. The procedure involves the use of the oxazaborolidinium ion as a Lewis acid catalyst; this species was generated from the corresponding oxazaborolidine by protonation with triflic imide or triflic acid (Figure 8). In previous studies, this class of catalysts was successfully employed in the enantioselective cyclopropanation of α-substituted acrolein with diazoacetates [62,63].

Based on this evidence, Ryu and coworkers showed that by reacting benzyl diazoester with benzaldehydes in the presence of 20 mol % oxazaborolidinium ion (activated by triflic acid—**4a**), optically active α-alkyl-β-ketoester could be efficiently prepared (compounds **6**—Table 1). Although the phenyl migration, yielding compounds **7,** could not be fully suppressed, this undesired pathway could generally be well controlled.

By modifying chiral catalysts and solvents, the chiral catalyst **4e** in toluene at −78 °C provided optimal reaction conditions in terms of chemoselectivity, enantioselectivity, and yield, thus resulting in the optically active α-alkyl-β-ketoester, with a 94:6 ratio, 95% ee, and 92% yield (Table 1, entry 7).

**Figure 9:**
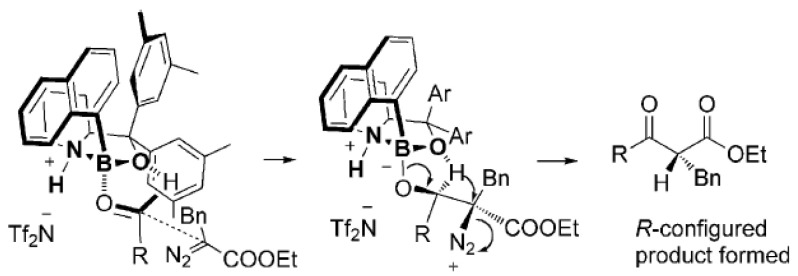
Transition state model for the reaction of α-benzyl diazoester with propionaldehyde in the presence of **4b**.

**Figure 10:**
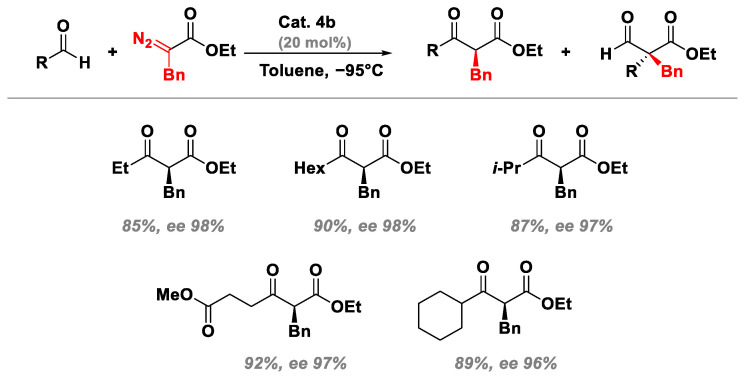
Application of aliphatic aldehydes to the novel asymmetric Roskamp reaction in the presence of the catalyst **4b**.

A diverse set of substituted benzaldehydes was tested, allowing the reaction to proceed in a highly chemoselective manner (Table 2). Further investigation of the substrate scope, particularly in order to extend the procedure to aliphatic aldehydes, showed poorer enantioselectivity when **4e** was used. On the other hand, the reaction catalyzed by **4b**—bearing the bulkier naphthyl group on the boron—allowed for increased enantioselectivity (Figure 9). This latter catalyst has been previously employed in enantioselective cyanosilylations, 1,3-dipolar cycloadditions, and cyclopropanations [63,64]. Presumably, the coordination of the aldehyde it exerts justifies the observed stereocontrol (Figure 10) [65].

The 3,5-dimethylphenyl group of **4b** shields the *Re* face (back) from the attack of the diazoester, which, due to the dipole–dipole interaction between the two carbonyl groups, approaches the aldehyde with the ester group situated away from the aldehyde group. As a result of steric interactions, the benzyl group is positioned away from the aldehyde alkyl group.

Thus, nucleophilic addition on the *Si* face (front) is facilitated, allowing for chemoselective hydride transfer with the loss of nitrogen in the intermediate state, forming the (*R*)-product as the major enantiomer. This mode of interaction gives catalyst **4e** a higher *ee* value compared to catalyst **4d** (Table 1).

**Figure 11:**
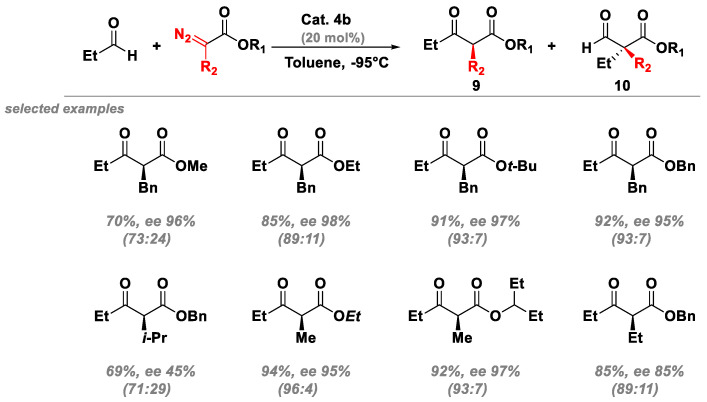
Application of various α-alkyl diazoesters to the novel asymmetric Roskamp reaction in the presence of the catalyst **4b**.

The catalytic system with **4b** was also applied to various α-alkyl diazoesters for the implementation of the methodology with propionaldehyde (Figure 11). While more sterically hindered R_1_ groups resulted in enhanced selectivity, sterically hindered R_2_ substituents caused a significant decrease in the ratio of **9/10** and enantioselectivity.

### 3.4. The Roskamp Reaction for Enantioriched Weinreb Amides

**Figure 12:**
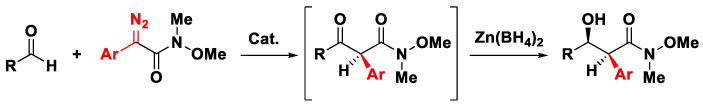
Roskamp reaction for enantioriched Weinreb amides.

Based on the concept that Weinreb amides have emerged as excellent acylating agents for carbanions [66,67,68], as well as in asymmetric transformations [69,70], Ryu synthesized a variety of highly optically active *syn*-α-aryl-β-hydroxy Weinreb amides via a one-pot procedure [71] (Figure 12), thus overcoming the limited substrate scope and low yields of previously reported procedures for the synthesis of α-aryl-β-hydroxy carbonyl compounds [72,73,74].

First attempts involving ethyl or *tert*-butyl α-phenyl diazoesters with *p*-tolualdehyde in the presence of 20 mol % oxazaborolidinium ion **13a**—activated with triflic amide at −40 °C in toluene—resulted in the formation of the *p*-tolyl migration product **12** in ∼60% yield (Table 3, entries 1 and 2). Changing the ester functionality of the diazo compounds to Weinreb amides afforded the α-phenyl-β-keto Weinreb **11** as the major product, with 80% *ee* and 47% yield in an excellent **11/12** *ratio*. Unfortunately, to avoid the erosion of chiral information, chromatography had to be carried out at −78 °C.

Further investigations on the effect of different substituents on the boracycle catalyst indicated that the 9-phenanthrenyl group was the best one (Table 3, entry 7), yielding the α-phenyl-β-keto Weinreb amide **11b** in 90% yield and 91% *ee*.

**Figure 13:**
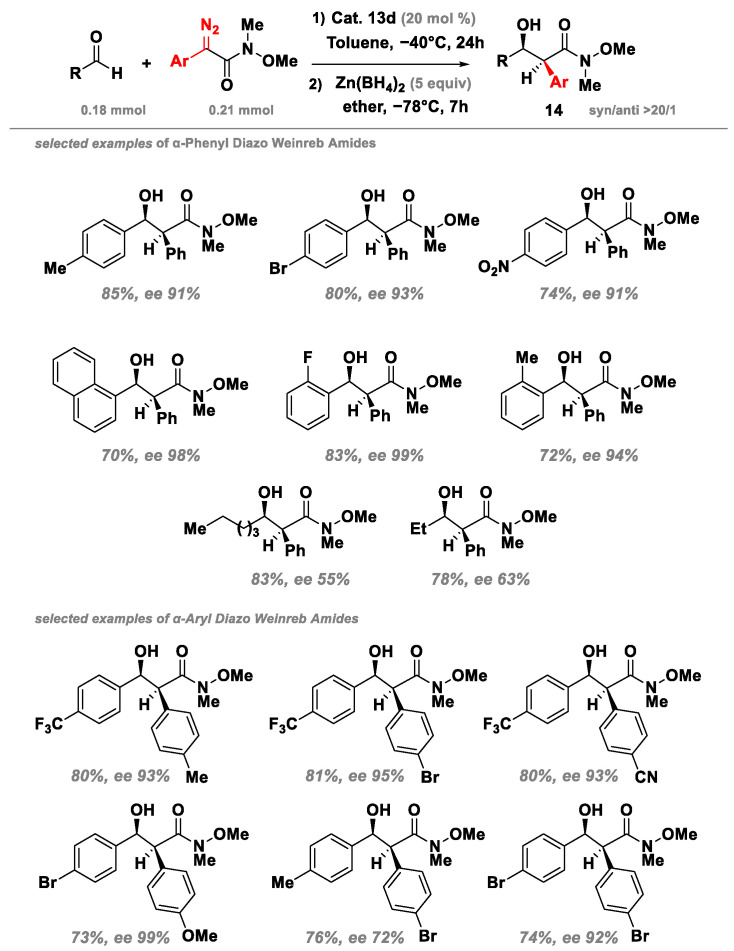
Asymmetric sequential Roskamp reaction/reduction reaction of α-phenyl diazo Weinreb Amides and α-Aryl diazo Weinreb amides with aryl aldehydes.

To overcome the difficulties in the isolation of the product at rt, a direct diastereoselective reduction with zinc borohydride at −78 °C was conducted, thus allowing for the preparation of *syn*-α-phenyl-β-hydroxy Weinreb amide **14** without the loss of optical purity. Accordingly, the desired products were obtained in good yields, high enantioselectivities, and excellent *syn* stereoselectivities, regardless of the electronic properties of the substituents on the aromatic aldehyde (Figure 13).

Investigating a variety of diazo Weinreb amides, the substitution at the para position with electron-withdrawing groups provided high enantioselectivities, while the installation of electron-donating groups on aromatic aldehydes resulted in lower enantioselectivities (Figure 13).

**Figure 14:**
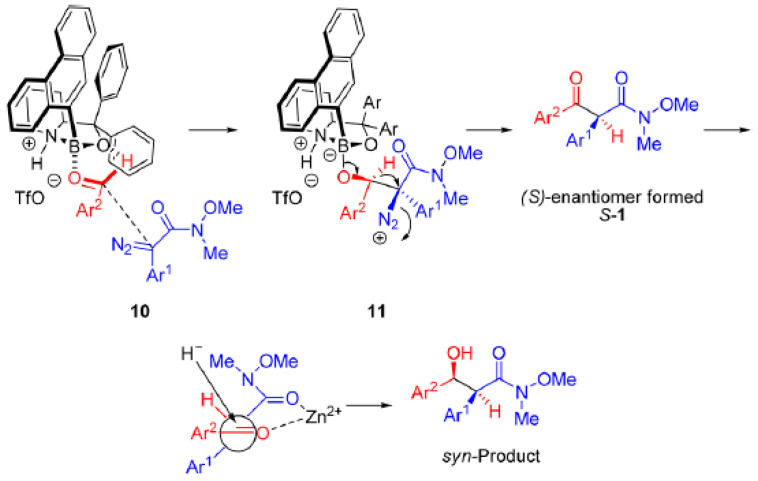
Model for the transition state in the asymmetric Roskamp reaction of α-Aryl diazo Weinreb Amides with aryl aldehydes catalyzed by **13d** and the reduction mechanism with zinc borohydride [58].

The observed stereochemistry using the oxazaborolidinium ion catalyst **13d** can be rationalized based on the transition state model shown in Figure 14. The mode of coordination of the aryl aldehyde to **13d** is the same as that previously observed in the enantioselective Roskamp reaction of diazoesters, and the *syn* stereochemistry of the product can be explained by the chelation-controlled model with Zn(BH_4_)_2_.

### 3.5. Roskamp Reaction of Silyl Diazoalkane

**Figure 15:**
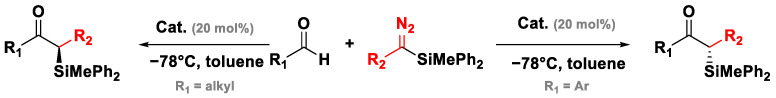
Catalytic asymmetric Roskamp reaction with silyl diazoalkanes.

The continuous quest for the development of diazomethane-free homologation methodologies prompted Ryu’s group to design a catalytic asymmetric Roskamp reaction using silyl diazoalkanes [75]. While trimethylsilyldiazometane was extensively used in various transformations [76,77], the use of silyl diazo alkanes was less explored. Thus, Ryu’s group envisioned that the use of these silylated reagents could provide a transition metal-free coupling approach to prepare valuable α-chiral silyl ketones (Figure 15) [78].

The Roskamp reaction between dimethyl phenylsilyldiazo ethane and benzaldehyde, in the presence of 20 mol % chiral oxazaborolidinium ion (COBI) **17a** activated by triflic acid, was carried out at −78 °C in toluene, yielding the desired optically active α-silyl ketone as the major product, with a 20% yield of the minor epoxide side product. Thus, screening the catalyst structure in the Ar_1_ and Ar_2_ substituents showed that 3,5-dimethylphenyl as Ar_1_ and 2-isopropoxyphenyl as Ar_2_ yielded the best results. The inversion of the configuration was observed when an *ortho* alkoxy-substituted phenyl group was employed as Ar_2_, and the introduction of one more phenyl group to the silyl diazoethane improved yield, enantioselectivity, and the 1**5/16** ratio (Table 4).

**Figure 16:**
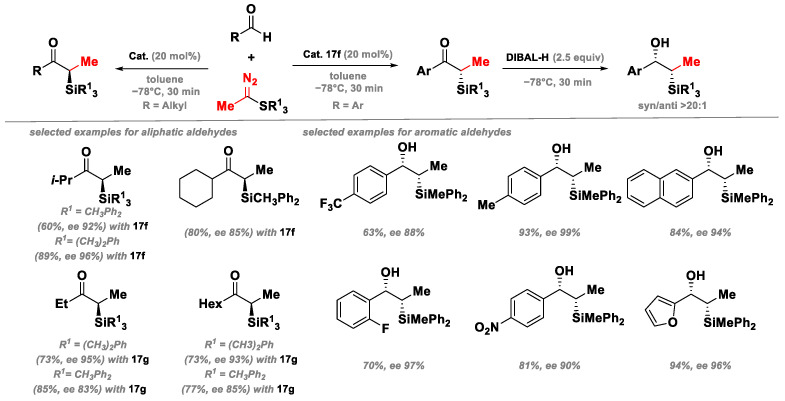
Asymmetric Roskamp reaction of silyl diazoalkane with aliphatic/aromatic aldehydes and consecutive reduction of the corresponding aromatic ketones.

Despite the fact that the protodesilylation on silica gel led to a considerable loss of yield, the development of a one-pot reduction of the corresponding ketones using the reduction with diisobutylaluminum hydride (DIBAL-H) at −78 °C yielded the desired products with good yield, high *ee* (>94%), and an excellent *syn/anti* ratio (>20:1) (Figure 16).

**Figure 17:**
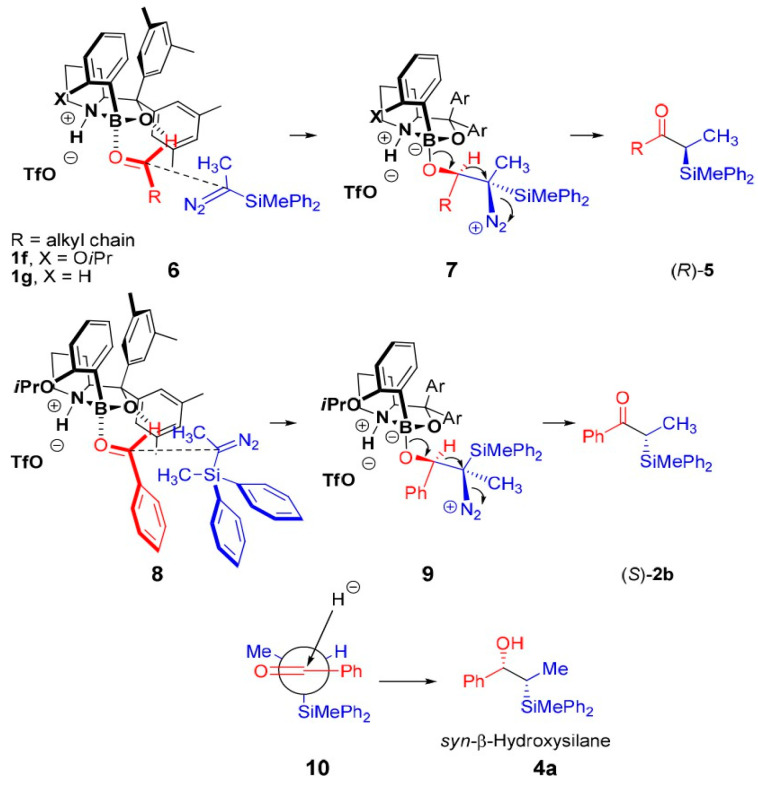
Transition state model for the asymmetric Roskamp reaction of silyl diazoalkane with aldehyde and the reduction mechanism with DIBAL-H.

The application of the methodology to aliphatic aldehydes, which are more stable during purification on silica gel, showed that the secondary aldehydes produced improved yields and enantioselectivity when additional phenyl groups were introduced on the silyl diazoethane. The best chiral oxazaborolidiniumion catalyst **17f** for aromatic and secondary aldehydes was found to be suboptimal for primary aldehydes; thus, it was replaced with catalyst **17g** (Figure 17). In contrast, the reaction of tertiary aldehydes was not feasible.

Using the transition state model shown in Figure 16, in which the coordination of the aldehyde to oxazaborolidinuim ion catalysts **17f** or **17g** follows the same pattern as previously postulated, the observed stereochemistry, (*R*) for aliphatic *α*-silyl ketone and (*S*) for the aromatic, can be explained. The stereochemistry of (*1S*,*2S*)-β hydroxysilane is explained using the Felkin−Anh model, in which the largest or bulkiest group at the α position prefers a conformation that is perpendicular to the plane of the carbonyl C=O group and anti to the incoming nucleophile, while the medium-sized group is placed *gauche* to the carbonyl.

## 4. The Roskamp Reaction in Total Synthesis

**Figure 18:**
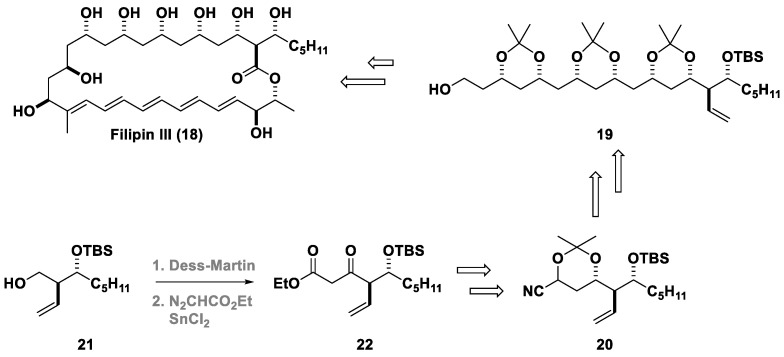
The Roskamp reaction in the total synthesis of Filipin III.

Natural products, with their structural complexity and medicinally relevant biological activities, play a pivotal role as prime targets in synthetic chemistry, inspiring the development of new strategies and the design of efficient methods to access substrates in larger amounts and more sophisticated structures. The construction of C-C single bonds represents one of the major focuses for accessing the unique carbon frameworks associated with these compounds, and in this context, the Roskamp reaction has proven its versatility in targeting a wide range of natural products. This strategy provided, for the first time, the total synthesis of filipin III (**18**), the major component of the filipin complex, a polyene macrolide antibiotic isolated from cell culture filtrates of *Streptomyces filipinensis.* The polyol segment of filipin III (**19**) was assembled starting from cyanohydrin acetonide **20**, synthesized via a mono-TBS protected diol **21**, which, upon oxidation to aldehyde followed by Roskamp’s procedure with ethyldiazoacetate, yielded the β-ketoester **22** in 81% yield, a pivotal building block for accessing the desired key fragment (Figure 18) [79].

**Figure 19:**
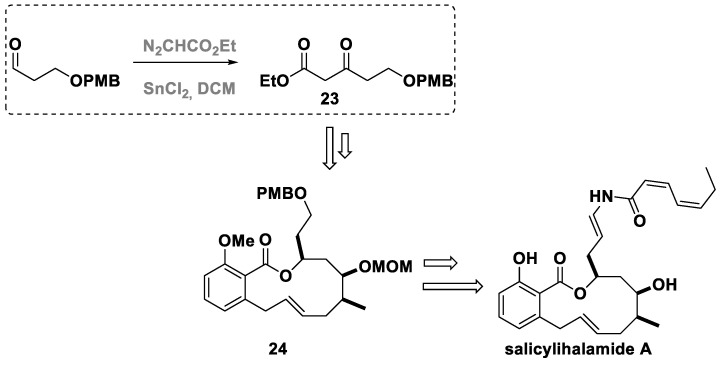
The Roskamp reaction in the total synthesis of Salicylihalamide A.

The chain extension tactic, employing ethyl diazoacetate in the presence of a catalytic amount of tin(II) chloride, was also adopted to access the 3-keto ester **23** for furnishing the macrolactone **24**. This represents a formal total synthesis of the benzolactone enamide salicylihalamide A (Figure 19), a potent and selective Vacuolar ATPase inhibitor, which is an enzyme involved in human cancer cells [80].

**Figure 20:**
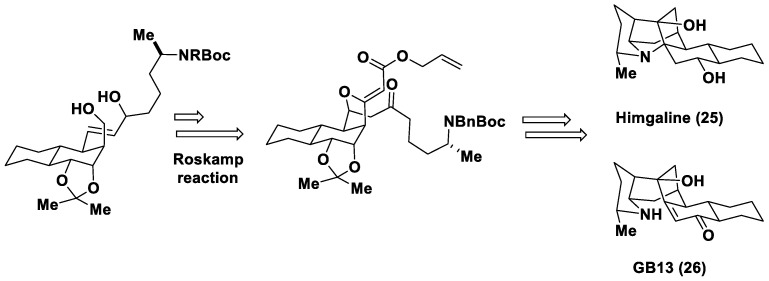
Access to *ent*-galbulimima alkaloid 13 and *ent*-himgaline via Roskamp reaction.

The molecular complexity of Himgaline (**25**) and galbulimima alkaloid 13 (GB13) (**26**), two members of a family of polycyclic alkaloids with potent muscarinic antagonist activity, has attracted the development of a synthetic strategy to access *ent*-galbulimima alkaloid 13 and *ent*-himgaline. The proposed pathway involves a selective Roskamp reaction to install the β-ketoester functionality to yield the final (+)-GB13, which, upon conjugate addition of the piperidine nitrogen into the proximal enone and subsequent stereoselective ketone reduction, furnished the targeted (+)-himgaline (Figure 20) [81].

The poor accessibility of natural compounds, often associated with their natural sources, prompted the development of more efficient synthetic routes to enable structure–activity relationship studies and facilitate target identification. With this aim, in 2012, *S. Gao* and coworkers reported the first asymmetric synthesis of (+)-fusarisetin A ((+)-1) [57].

**Figure 21:**
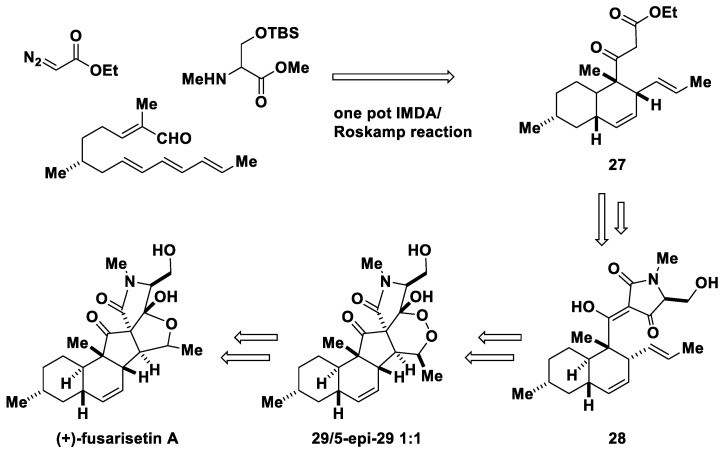
Asymmetric synthesis of (+)-fusarisetin A.

The strategy relies on a biosynthetic pathway involving a one-pot IMDA/Roskamp reaction to construct the *trans*-decalin skeleton **27**, followed by oxidation of equisetin **28** promoted by MnIII/O_2_. The IMDA reaction generates an aldehyde, which undergoes the Roskamp reaction in the presence of BF_3_·OEt_2_ in one pot, yielding **27** in 47% yield (Figure 21). The oxidation of **28** with Mn(OAc)_3_ in air or 1 atm of O_2_ at room temperature produces the peroxyfusaresetin **29** and 5-epi-**29** as inseparable diastomers, which, upon reduction with Zn/HOAc, furnish the 5-epi- and (+)-fusarisetin A in a 75% combined yield.

**Figure 22:**
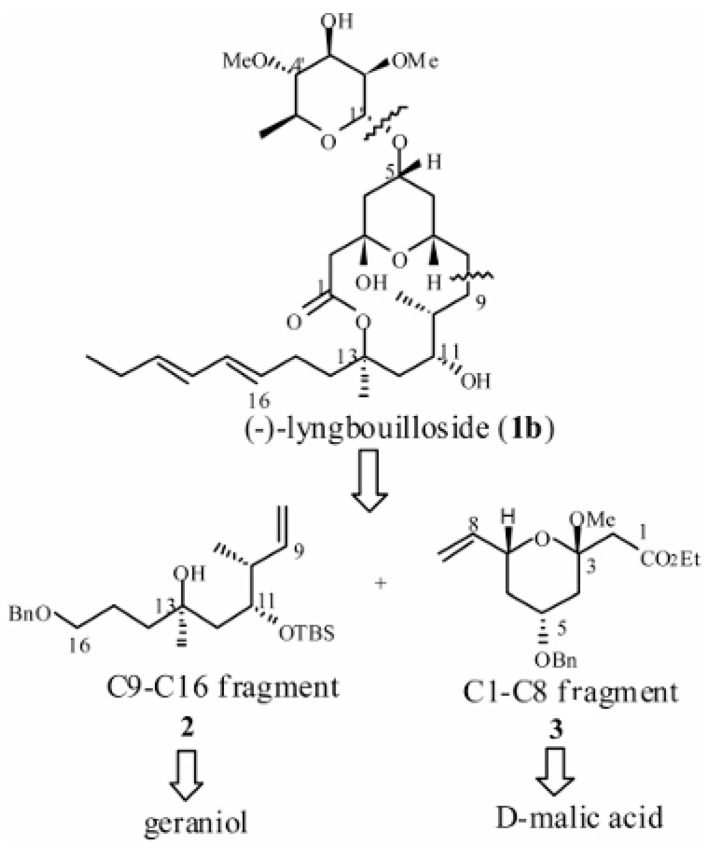
Retrosynthetic analysis of (−)-lyngbouilloside.

The structural complexity, along with the natural scarcity and biological properties of natural products, such as Lyngbouilloside, has attracted significant attention in the synthetic community. In this context, the Roskamp reaction found fertile ground for its applicability. In 2002, *Gerwick* and coworkers [82], reported for the first time the isolation of Lyngbouilloside (Figure 22), a novel glycosidic macrolide collected from the marine cyanobacterium *Lyngbya bouillonii*, present in Papua New Guinea and then found in New Britain. Although spectroscopic analysis allowed for the determination of the relative stereochemistry, the lack of available material and the complexity of the structure prevented the assignment of the absolute stereochemistry. These stereochemical ambiguities, along with the moderate cytotoxic activity toward neuroblastoma, prompted the synthetic community to address the challenge of accessing fragments of this nature.

Based on the work of *Cossy* et al. [83], which culminated in the stereochemical reassignment of the stereogenic center C11, establishing the absolute stereochemistry and revising the structure of the compound, *Yadav* and coworkers reported the synthesis of the C1–C8 and C9–C16 fragments of the revised structure of (−)-lyngbouilloside [84]. The restosynthetic approach leveraged the disconnection of the C8–C9 bond (Figure 22). The preparation of the C9–C16 fragment started with the commercially available geraniol.

**Figure 23:**
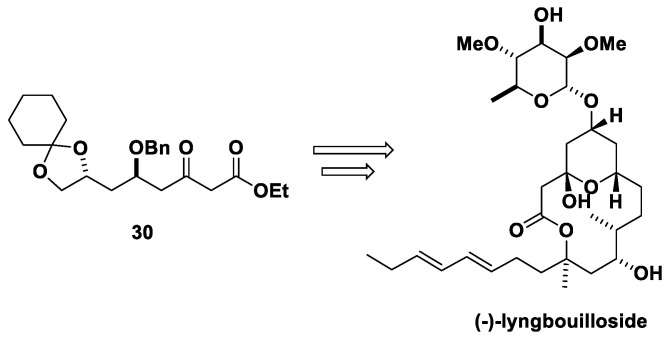
Synthesis of (−)-lyngbouilloside.

The synthesis of the C1–C8 fragment began with D-malic acid, which, upon oxidation, Keck allylation, the protection of the secondary hydroxyl group, and subsequent oxidative cleavage to aldehyde, underwent the Roskamp reaction with ethyl diazoacetate in the presence of a catalytic amount of tin(II) chloride, resulting in the formation of the β-keto ester **30** and leading to the desired fragment in 60% yield (Figure 23).

**Figure 24:**
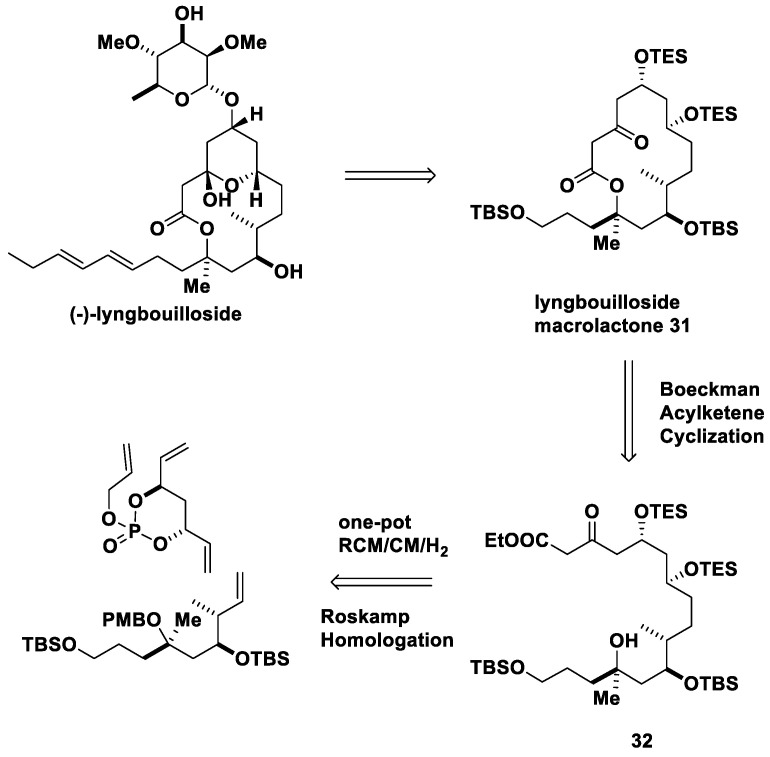
Retrosynthetic analysis of (−)-lyngbouilloside.

In 2015, *Hanson* and coworkers [58] reported a concise synthesis of the originally assigned macrolactone core **31**, which is easily adaptable to the C10/C11 and C13 diastereomeric analogs. The macrolactone core **31** was constructed through a one-pot phosphate tether-mediated, sequential RCM/CM chemoselective hydrogenation reaction, two-carbon Roskamp homologation, and a high-yielding Boeckman acylketene cyclization of the β-keto ester **32**. Pyrolysis of **32** in toluene under diluted conditions, with the azeotropic removal of EtOH, afforded **31** in excellent yield (90%) (Figure 24).

**Figure 25:**
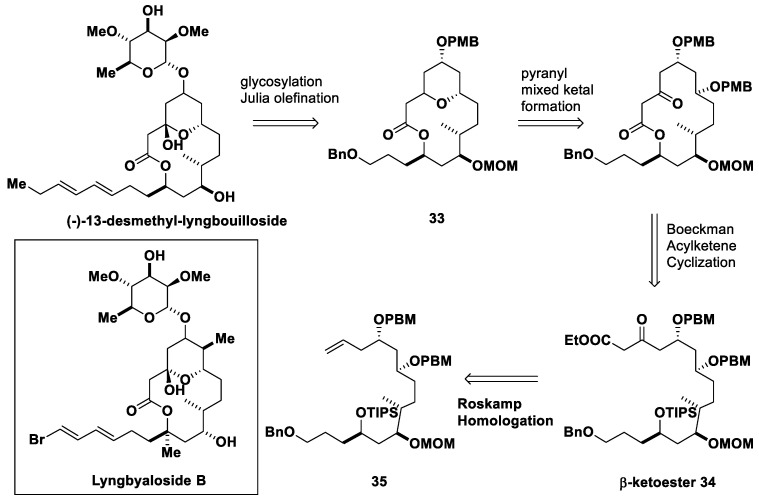
Retrosynthetic analysis of (−)-13-desmethyl-lyngbouilloside.

The same group, based on the work of *Fuwa* on the closely structurally related lyngbyaloside B [85], and motivated by the positive results in the total synthesis of (−)-lyngbouilloside [58], reported the asymmetric synthesis of (−)-13-desmethyl-lyngbouilloside and corresponding analogs (Figure 25). *Hanson* et al., employing a similar protocol reported for (−)-lyngbouilloside, accomplished the total synthesis of the targeted compound via a late-stage glycosylation and olefination of the mixed ketal macrolactone **33**, which can be accessed from the β-ketoester **34** upon Boeckman acylketene cyclization [59]. The synthetic pathway developed for **34** is based on the oxidative cleavage of the terminal olefin **35**, followed by a two-carbon Roskamp homologation, highlighting the application of the Roskamp reaction for the synthesis of the macrolactone core and more efficient access to these fragments.

## 5. Conclusions

From its discovery, the Roskamp reaction has proven to be an attractive methodology for accessing a series of α-β-carbonyl compounds, allowing for the reaction of α-diazo carbonyls with a variety of aldehydes in the presence of a Lewis acid. The ability of the reaction to be productive in a mild environment, along with the beneficial coordination of the reactants with a variety of Lewis acids and catalysts, opens the door to the development of related procedures aimed at improving the feasibility of the process in more environmentally friendly settings (e.g., applicability in continuous-flow systems) and accessing challenging chiral substrates. The significant interest in modern homologative chemistry to access enantioenriched α-β-functionalized compounds, bearing reactive functionalities such as Weinreb amides or silyl alkanes, along with the challenge of synthesizing natural compounds more efficiently, highlights the ongoing applicability of the Roskamp reaction.

## Figures and Tables

**Table 1:**
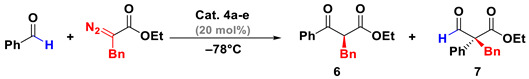
Optimization of the oxazaborolidinium ion Lewis acid-mediated asymmetric Roskamp reaction.

Entry	Solvent	Cat.	Time [h]	6/7	%Yield	*ee* [%]
1	DCM	**4a**	1	90:10	82	87
2	DCM	**4b**	1	89:11	80	90
3	DCM	**4c**	1.5	82:18	77	82
4	Propionitrile	**4c**	1	67:33	60	79
5	Toluene	**4c**	1.5	91:9	87	88
6	Toluene	**4d**	1	93:7	90	92
7	Toluene	**4e**	2	94:6	92	95

**Table 2:**
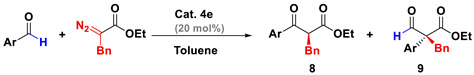
Application of aromatic aldehydes to the novel asymmetric Roskamp reaction in the presence of the catalyst **4e**.

Entry	Ar	T(°C)	Time [h]	8/9	%Yield	*ee* [%]
1	Ph	−78	2	94:6	92	95
2	4-MeC_6_H_4_	−78	2	90:10	87	95
3	4-MeOC_6_H_4_	−55	1	92:8	85	91
4	4-BrC_6_H_4_	−78	1	94:6	91	94
5	4-CF_3_C_6_H_4_	−95	1	89:11	82	96
6	3-OMe	−78	2	90:10	83	95
7	2-OMe	−55	5	76:24	69	80

**Table 3:**
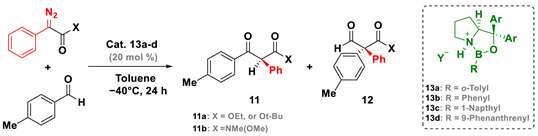
Optimization procedure for the asymmetric Roskamp reaction of α-phenyl diazo Weinreb amides with *p*-tolualdehyde.

Entry	Cat.	X	Y	11/12	%Yield	*ee* [%]
1	**13a**	OEt	Tf_2_N	1:3	22	-
2	**13a**	*t*-BuO	Tf_2_N	1:3	20	-
3	**13a**	NMe(OMe)	Tf_2_N	>20:1	47	80
4	**13a**	NMe(OMe)	TfO	>20:1	51	82
5	**13b**	NMe(OMe)	TfO	>20:1	92	89
6	**13c**	NMe(OMe)	TfO	>20:1	87	91
7	**13d**	NMe(OMe)	TfO	>20:1	90	91

**Table 4:**
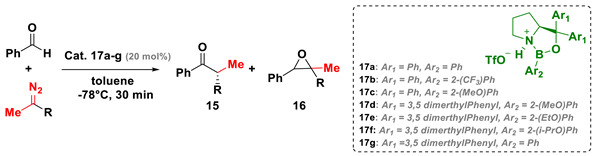
Optimization procedure for the reaction of silyl diazoethane (0.28 mmol) with benzaldehyde (0.23 mmol).

Entry	Cat.	R^1^	15/16	%Yield	*ee* [%]
1	**17a**	(CH_3_)_2_PhSi	2:1	41	−34
2	**17b**	(CH_3_)_2_PhSi	6:1	40	−50
3	**17c**	(CH_3_)_2_PhSi	4:1	62	68
4	**17d**	(CH_3_)_2_PhSi	5:1	68	81
5	**17e**	(CH_3_)_2_PhSi	4:1	63	92
6	**17f**	(CH_3_)_2_PhSi	9:1	73	92
7	**17g**	CH_3_Ph_2_Si	>20:1	79	98

## Data Availability

No new data were created or analyzed in this study. Data sharing is not applicable to this article.
